# Patient recall of receiving lifestyle advice for overweight and hypertension from their General Practitioner

**DOI:** 10.1186/1471-2296-11-8

**Published:** 2010-02-01

**Authors:** Alison O Booth, Caryl A Nowson

**Affiliations:** 1 Nutrition and Metabolism Research Group, Queens University Belfast, UK; 2 The Centre for Physical Activity and Nutrition, School of Exercise and Nutrition Sciences, Deakin University, Burwood, Australia

## Abstract

**Background:**

Overweight, obesity and hypertension can be prevented through improvements in lifestyle including nutrition and physical activity. General practitioners (GPs) in Australia have access to over 90% of the population in the course of a year and therefore, the general practice setting may be ideal to assist patients with lifestyle change for weight management and hypertension. The present study aimed to determine the proportion of overweight/obese patients that recalled receiving advice by their GP to make lifestyle changes for weight loss. Recall of advice received by hypertensive patients to reduce salt intake was also measured.

**Methods:**

A face to face survey was conducted on a representative sample (urban, suburban and rural) of South Australian residents. Respondents provided information on height and weight (self-report), whether they had received lifestyle advice from their GP for weight loss, and for those with self reported hypertension if they had received advice to reduce dietary salt.

**Results:**

The sample included 2947 South Australian adult residents (58% female; BMI (mean (SD)), 26.6 (5.3) kg/m^2^; age, 50.7 (18.0) years). Ninety-six percent had visited their GP in the past 12 months. Forty-one percent of males and 25% of females were overweight and 19% of males and 20% of females were obese. Twenty-seven percent of overweight/obese respondents reported receiving lifestyle advice for weight loss purposes. Of the 33% who reported they had hypertension, 34% reported receiving advice to reduce salt intake.

**Conclusions:**

Less than 1/3 of overweight/obese patients reported that they had received lifestyle advice that could assist with weight loss from their GP. About a third of respondents with hypertension reported that they received advice to reduce salt intake. There are potentially missed opportunities in which GPs could provide re-enforcement of benefits of lifestyle changes with respect to weight and blood pressure control.

## Background

Ischaemic heart disease and strokes are the leading causes of death in Australia accounting for 24% of total deaths in 2007[1]. Two of the major risk factors for heart disease and strokes are hypertension and a high body mass index. In Australia, 42% of males and 31% of females were overweight and 26% of males and 24% of females were obese in 2007-08[2]. In 2007-08, 9% of all Australian adults and 39% of those aged over 75 years had high blood pressure[2].

Overweight, obesity and hypertension can be prevented through improvements in lifestyle including nutrition and physical activity. There is a clear association between excessive energy intake and low levels of physical activity and overweight/obesity. In addition, higher dietary salt intake is associated with hypertension[3] and a reduction in dietary salt reduces blood pressure[4]. A reduction in salt intake on a population level has been predicted to reduce the risk of stroke and cardiovascular disease and considerably reduce health care costs[5].

In recent years, there has been a research-based focus on using the general practice setting for health promotion including improving dietary and exercise practices among patients, particularly for weight management. In 2000, general practitioners (GPs) in Australia had access to over 90% of the population in the course of a year[6] and therefore, may be an ideal setting to assist with weight management and hypertension. Although there is limited evidence in the area, GPs have been found to be effective in improving health behaviours, including increasing physical activity[7] and improving nutrition[8]. There is also evidence that patients who receive advice to eat less fat or exercise more from their GP, are more likely to remember printed materials on the same topics and report making changes in diet and/or activity than those who did not receive the advice from their GP[9]. However, such printed materials are not readily available in Australian general practice and one study highlighted that when printed materials were made available, they were used infrequently[10]. Additionally, only a few studies have investigated lifestyle advice specifically for weight management or for the management of hypertension. Some interventions have shown positive results, however these are often time-intensive[11].

Australian GPs are recommended to follow guidelines for the management of overweight[12] and hypertension[13]. The 10 step guide to help manage overweight includes prescribing or referring for dietary and physical activity advice, in most cases before prescribing medication or surgery. The hypertension guidelines state 'Lifestyle modification is indicated for all patients with hypertension, regardless of drug therapy'[13]. Therefore, it is clear that lifestyle is a priority for GPs to manage overweight and hypertension in Australian practices.

Data indicates that hypertension is the most frequently managed problem in general practice in Australia and that 3.4 of every 100 encounters involved nutrition or weight management advice[14]. However, there is limited research that has examined the frequency Australian GPs give lifestyle advice to patients for weight loss or hypertension.

The aims of this project were; to determine the proportion of overweight/obese patients that recalled receiving advice by their GP to make lifestyle changes for weight loss and to determine if there was any gender bias in the recall of advice; to determine the proportion of hypertensive patients that recall receiving advice from their GP to reduce intake of salt and/or salty foods and; to determine the proportion of overweight/obese patients with hypertension who recall receiving lifestyle advice compared to patients who were overweight/obese without hypertension.

## Methods

### Health Omnibus Survey

The Health Omnibus Survey (HOS) is an annual survey executed by Harrison Health Research in South Australia. The survey consists of questions (selected by several organisations) relating to the health and wellbeing of South Australian residents and the purpose is to provide these organisations with the opportunity to generate data that can be used to plan, implement and/or monitor various programs and initiatives. The whole survey is designed to take approximately 30 minutes to complete. Deakin University placed 9 questions on this survey relating to lifestyle advice GPs offer to patients and their practices relating to this advice. Standard demographic questions were also collected. The survey was executed between September and October, 2005.

The survey included 10 demographic questions (includes gender, age, gross annual household income, marital status, highest educational qualification obtained, country of birth, length of time living in Australia (for those born outside of Australia), Aboriginal/Torres strait Islander status, occupation (what kind of work you have done for most of your life) and postcode (data not shown). An additional 9 questions were placed on this survey. The questions asked for self-reported height and weight, whether they have received dietary or exercise advice from their GP for weight loss, whether their GP has ever recorded their weight, whether their GP has referred them on to another party for weight loss purposes, if they had ever been told by a doctor that they had high blood pressure, if they currently take medication for the control of blood pressure and if they have ever received advice from their doctor to reduce their intake of salt and/or salty foods. The survey did not ask respondents to provide any further details regarding diet or exercise advice (including time spent, type of advice), just wether they recalled receiving it or not. From self reported height and weight, BMI was calculated and respondents were placed in BMI categories according to the World Health Organisation (WHO) classifications; underweight < 18.5 kg/m^2^, healthy weight 18.5 - 24.9 kg/m^2^, overweight 25 - 29.9 kg/m^2^, and obese ≥ 30 kg/m^2^. The small percentage of missing data for BMI category included those who could not recall their weight and/or height.

### Sample size and selection

A sample of 5000 households was selected. Seventy seven percent of the sample was from cities and towns with a population size of 10,000 or more, with the remainder from country areas in South Australia that have a population of 1000 or more. Smaller towns than this are too remote and therefore add too much to the cost of the survey. South Australia is divided into ABS Collection Districts (CDs) comprising of approximately 200 dwellings each. Three-hundred and eighty-six of these CDs were selected to participate in the survey. Ten households were selected from each of 386 CDs. A randomly selected starting point with fixed skip intervals selected the CDs for participation. For example, from the randomly selected starting CD, every 5^th ^(or other nominated number) CD from that point was selected. The ten households within each CD were also selected using the same fixed skip interval technique from a random starting point. Within each household the person who was last to have a birthday (aged 18 or over) was selected to participate. If the selected participant was not available, then the interview was not conducted on alternate household members.

### Statistical analyses

All statistical analysis was performed using SPSS for Windows (SPSS Inc., Chicago, Illinois, release 14.0.2, Chicago). Age and BMI were normally distributed and therefore, parametric analyses were used. Multivariate ANOVA was used to analyse differences in anthropometric measurements between genders and the difference in advice received by BMI. Pearson chi square was used to; determine differences between BMI categories and the frequency of advice received; differences between BP status and advice received and; differences between the frequency of advice received and age, gender, education and income. For this, age was categorised in 6 groups (18-24, 25-34, 35-44, 45-54, 55-64 and 65+ years), education was categorised in 5 groups (Bachelor degree or higher, certificate/diploma, trade/apprenticeship, left school after age 15 years or still studying, and left school before age 15 years) and income was categorised into 4 groups (up to 20,000, 20,001 - 40,000, 40,001 - 60,000 and 60,000 AUS$ or more). A multi-variate analysis was used to assess whether age and BMI were determinants of advice received.

### Ethical approval

This study was approved by the Deakin University Human Research Ethics Committee and the South Australian Department of Health's Human Research Ethics Committee.

## Results

### Sample

Of an initial sample of 5,000 households drawn from 386 CDs across South Australia, 173 were vacant, businesses, or not permanent tenants. Of the remaining 4827 houses, a total of 3047 interviews were conducted. Reasons for not participating include refusal to participate (n = 1011), not contactable after 6 visits (n = 525), respondent unable to speak English (n = 70), respondent away for the duration of the survey (n = 65), illness or incapacity (n = 72), interviewer unable to gain access to building (n = 32) and ferocious dog (n = 3). The overall response rate was 63.1% and the participation rate (excluding those non contactable after 6 attempts) was 70.8%. The four questions covered in this paper (the questions apart from weight and height) were relevant only to those who had visited their GP in the past 12 months. Ninety-six percent of respondents visited their GP in the previous 12 months. It is this 96% that are included in the analysis. One hundred and seven (3.6%) who did not provide details on height and 160 (5.4%) who did not provide details on weight were excluded from analysis.

### Respondents

The questions for the present study were only asked to survey respondents who were 18 years of age or older. The final study sample reported in this paper consists of 2947 respondents 18 years of age or older (58% female). Characteristics of respondents are presented in table 1.

**Table 1:** Characteristics of participants (95% Confidence Interval)

	Males n = 1239	Females n = 1708
Age (years) †	50.8 (49.8, 51.2)	50.7 (49.8, 51.5)
**Marital status (%)**		
- Married/de facto (living together as a couple)	61.6 (58.9, 64.3)	56.3 (54.0, 58.7)
- Separated/divorced/widowed	17.2 (15.1, 19.3)	28.9 (26.8, 31.1)
- Never Married	21.1 (18.8, 23.3)	14.8 (13.1, 16.5)
**Highest education level(%)**		
- Bachelor degree or higher	16.3 (14.2, 18.4)	16.7 (14.9, 18.5)
- Certificate/diploma	18.5 (16.3, 20.6)	28.0 (25.9, 31.1)
- Trade/apprenticeship	25.7 (23.2, 28.1)	3.9 (3.0, 4.8)
- Left school after age 15 years or still studying	24.8 (22.3, 27.8)	32.5 (30.3, 34.7)
- Left school before age 15 years	13.7 (11.8, 15.6)	18.3 (16.4, 20.1)
**Annual household income (AUD) (%)**		
- up to 20,000	18.2 (16.0, 20.3)	26.0 (23.9, 28.1)
- 20,001 - 40,000	21.9 (19.6, 24.2)	20.0 (18.1, 21.9)
- 40,001 - 60,000	18.3 (16.2, 20.5)	17.0 (15.2, 18.8)
- 60,000 or more	32.7 (30.1, 35.3)	26.3 (24.2, 28.4)
Weight (kg) †	84.2 (83.3, 85.0)	69.9 (69.1, 70.6)**
Height (cm) †	177.0 (176.6, 177.4)	163.0 (162.7, 163.4)**
BMI (kg/m^2^) †	26.9 (26.6, 27.2)	26.3 (26.1, 26.6)
BMI Categories (%)		
- Underweight (BMI < 18.5)	1.0 (0.5, 1.6)	3.0 (2.2, 3.8)**
- Acceptable weight (BMI 18.5 - 24.9)	32.9 (30.3, 35.5)	41.5 (39.2, 43.8)**
- Overweight (BMI 25 - 29.9)	41.5 (38.8, 44.2)	25.8 (23.7, 27.9)**
- Obese (BMI ≥ 30)	19.0 (16.8, 21.2)	20.1 (18.2, 22.0)
- Missing‡	9.6 (8.0, 11.2)	5.6 (4.5, 6.7)**
High blood pressure (%)	29.5 (27.0, 32.0)	34.8 (32.5, 37.1)**
- On medication (%)	69.0 (64.3, 73.7)	65.3 (61.5, 69.1)
- Advised to reduce salt intake (%)	37.5 (32.5, 42.5)	31.0 (27.3, 34.7)

†Mean (95% Confidence Interval).

‡Missing - did not report height or weight.

**p < 0.01 (difference between sexes)

### BMI

Nearly 42% of males and 26% of females were overweight (difference, *p *< 0.001). Nineteen percent of males and 20% of females were obese (Table 1). Overweight and obese respondents were older (51.8 ± 16.1(SD) years) compared to healthy/under weight (2.2% of respondents were underweight) respondents (48.8 ± 19.4 years) (*p *< 0.001) and were more likely to be married (*p *< 0.01). Of those who were overweight/obese, 46% were weighed in the previous 12 months by their GP.

### Lifestyle advice for weight loss

Fifteen percent of overweight and obese respondents reported receiving both diet and exercise advice for weight loss, 5.5% reported receiving only dietary advice, 6.5% reported receiving only exercise advice and 6% were reported being referred elsewhere (Table 2). Overall, 17% of respondents reported they received some form of lifestyle assistance for weight loss from their GP (dietary or exercise). Obese respondents were more likely to report that they received dietary advice, exercise advice and be referred elsewhere than overweight respondents (*p *< 0.001, Pearson Chi square).

**Table 2:** Frequency (%) overweight patients reported receiving advice from their GP for weight loss, with and without hypertension.

	n (%)	Diet only†† %	Exercise only‡ %	Diet plus exercise ¶ %	Referral§ %	Diet, exercise and referral ‡‡ %	Any advice or referral §§ %	Salty foods ¶¶ %
Healthy weight/underweight	1180 (38.7)	2.0	0.8	1.9	0.7	0.3	13.3	9.9
Overweight and obese	1534 (50.3)†	5.5†	6.5†	15.1†	6.3†	4.2†	38.0	19.0†
Overweight (BMI 25-29.9 kg/m^2^)	955 (31.3)	3.7^	4.7^	8.6^	3.2^	1.9^	30.3	17.9
Obese (BMI ≥ 30.0 kg/m^2^)	579 (19.0)	8.6	9.3	25.7	11.2	8.1	50.8	20.9
Overweight & hypertensive	600 (19.7)	7.8*	8.7*	20.7**	9.0**	6.5**	57.5	37.5**
Overweight without hypertension^^	928 (30.5)	4.1	5.0	11.5	4.5	2.8	25.5	7.2
Healthy weight/underweight & hypertensive	275 (9.0)	4.4	2.9	2.9	1.8	1.1	33.8	28.4

*difference from 'overweight without hypertension' group, P < 0.01, Pearson Chi Square

**difference from 'overweight without hypertension' group, P < 0.001, Pearson Chi Square

†difference from the 'healthy weight/underweight' group, P < 0.001, Pearson Chi Square

^difference from the 'obese (BMI <30 kg/m^2^)' group, P < 0.001, Pearson Chi Square

††Respondents who reported receiving only dietary advice but not exercise advice for weight loss from their GP in the previous 12 months.

‡Respondents who reported receiving only exercise advice but not dietary advice for weight loss from their GP in the previous 12 months.

¶Respondents who reported receiving both diet and exercise advice for weight loss from their GP in the previous 12 months.

§Respondents who reported they were referred elsewhere for weight loss by their GP in the previous 12 months.

‡‡Respondents who reported they received both exercise and dietary advice for weight loss from their GP and were referred elsewhere

§§Respondents who have received any advice (exercise, diet or advice to reduce salt intake) or were referred elsewhere by their GP in the previous 12 months.

¶¶Respondents who reported receiving advice to reduce their intake of salt or salty foods from their GP in the previous 12 months.

^^'without hypertension' refers to those who did not report having hypertension or report being on medication for hypertension.

There was no difference in self reported GP lifestyle advice by patient gender, education or income (Pearson Chi Square, data not shown).

### Dietary advice for blood pressure

Approximately a third of all respondents reported that they had high blood pressure and 21% were taking medications for blood pressure control. Of those who reported they had high blood pressure, two thirds were taking medication (for blood pressure control). More females than males had been told by their doctor in the past that they had high blood pressure. Just over a third of those who reported they had hypertension had been advised to reduce salt intake by their doctor (Table 1). Patients with hypertension who were also taking blood pressure medication were more likely to recall receiving advice to reduce the intake of salt and salty foods (43%) than persons with hypertension that were not taking medication (16%) (P < 0.001, Pearson Chi Square).

### Lifestyle advice to patients with overweight/obesity and hypertension

Overweight/obese respondents with hypertension were nearly twice as likely to report receiving lifestyle advice and five times more likely to report receiving advice to reduce salt intake from their GP than overweight/obese without hypertension respondents (Table 2).

Respondents who reported receiving exercise or dietary advice had a higher BMI than those who did not (exercise, 32.0 ± 0.3 (SEM) kg/m^2 ^versus 25.5 ± 0.4 kg/m^2^, *p *< 0.01; diet, 31.3 ± 0.3 (SEM) kg/m^2 ^versus 25.8 ± 0.1 kg/m^2^, *p *< 0.01). Respondents who reported receiving exercise advice were younger than those who reported that they did not receive exercise advice (52.3 ± 1.0 years versus 59.3 ± 1.5 years, *p *< 0.01) but there was no difference in age among those who reported receiving dietary advice (*p *= 0.219). Neither age nor BMI correlated with receiving advice to reduce salt intake.

## Discussion

### Lifestyle advice for weight loss

This study in almost 3,000 South Australian residents indicated that less than a third of overweight and obese respondents reported they received dietary and/or exercise advice for weight loss in the past 12 months from their GP. Similar findings have been reported in the US[15] where39% of obese adults who had visited their GP in the previous 12 months were advised to lose weight. The rates of obesity and overweight in our study were similar to a nation-wide representative sample of patients visiting their GP (BEACH) (self report)[14]. GPs may not offer lifestyle advice as they view it as ineffective, although GPs appear to agree that nutrition is important in managing disease[16]. GPs often face many barriers to offer lifestyle advice to patients, particularly if the presenting problem is unrelated. GPs may lack confidence in offering more detailed nutrition advice[17] due in part, to insufficient knowledge[18]. Other barriers include time constraints, a lack of incentives or reimbursements[19], complexity of advice, lack of training in counselling skills, a lack of interest[20], the idea that patients are not motivated[17] and a long delay between intervention and observable effects[21].

Of every 100 encounters in Australian general practice, 9.4 involve managing hypertension[14]. Additionally, of all conditions, 3.4 of every 100 encounters involved nutrition or weight counselling[14]. However, the frequency does not match the fact that the majority of patients are overweight or obese (nearly 60%) with only 0.6 in 100 encounters involving treatment for obesity. However, these findings are difficult to compare to the findings in the present study owing to the fact that the question in the present study specifically referred to 'diet advice for weight loss' and the above mentioned study referred to non-specific nutrition or weight counselling. In addition, patients may recall receiving small pieces of advice from their GP in our study but the 'counselling' may refer to more in depth advice.

Although GPs may consider that lifestyle advice will not be well received by patients, a survey found that nearly 80% of general practice patients in NSW agreed that GPs should have a role in weight management[22]. There is also evidence that patients are more likely to recall lifestyle advice and attempt to make lifestyle changes when they receive that advice from their GP[9].

### Blood pressure and advice to reduce salt intake

Just under a third reported that they had ever been told by a doctor that they had high blood pressure and around 20% were taking blood pressure medication. This is higher than the current Australian prevalence rate among adults (9%) and may be due, in part, to the older age group in this study and/or the survey question design. Our study also found that around a third of patients who had been told they had high blood pressure reported receiving advice to reduce salt intake. This figure is slightly lower than a study in the UK (40%)[23] that was conducted before the media messages to reduce UK salt intake[24].

### Lifestyle advice for patients with overweight/obese and hypertension

Overweight/obese respondents with hypertension were more likely to report receiving dietary and exercise advice from their GP than overweight/obese respondents without hypertension. This supports other findings that GPs are more likely to offer such advice to patients with multiple risk factors than ones with fewer risk factors[10]. Despite this, the rate of advice was still low (less than 30%). The general practice setting has traditionally been treatment focussed, therefore it appears that GPs prefer to use nutrition intervention for treatment to patients who are viewed to be in greater need for intervention[25].

### Strengths and Limitations

The 67% response rate in our study may have been limited due to the sampling procedure whereby if the person with the next upcoming birthday was not available, then no other household members could be approached. Self reported height tends to be an overestimate and weight an underestimate[26], which means we may have underestimated the rates of overweight/obesity. Additionally, accuracy in recall of lifestyle advice is dependent on memory, which may be variable and it's possible that this survey may have underestimated GP practices. However, results from a previous study suggests that such underreporting by respondents is unlikely[27] and if patients do not recall receiving advice it probably is of no benefit[28]. However we could make no assessment of the quality and quantity of lifestyle advice received, only patient recall of receiving advice. Data on the frequency of GP visits was not obtained which could have affected the number of opportunities respondents had in receiving advice.

Within this research, it is unknown what proportion of consultations were pre-scheduled reviews of hypertension or obesity or in how many consultations did the patient request the advice. Additionally, GPs in Australia on average conduct 108 consultations each week full time and a usual consultation lasts around 12 minutes[29]. This does not leave much room available for a discussion on health promotion or nutrition, particularly if the presenting problem is unrelated or if no reimbursement is forthcoming.

It is possible that those with hypertension received additional lifestyle and dietary advice from their GP that did not include the reduction of salt and salty foods. Due to the limited number of questions placed on the survey, we were not able to ask about additional lifestyle advice that may be important in the management of hypertension. However, reducing salt intake is one simple piece of advice that has the potential to make a difference on an individual level as well as a population level[4,5] if achieved.

## Conclusions

Less than a third of overweight and obese patients in Australia reported receiving lifestyle advice from their GP. Few overweight and obese patients reported being referred elsewhere for weight loss purposes by their GP which may be due, in part, to an insufficient number of appropriate health professionals such as dietitians available in the community public sector. There are potentially many missed opportunities in which GPs could provide lifestyle counselling for weight management.

Hypertension being the most frequently managed problem in Australian general practice[14] highlights the opportunities available to offer nutrition advice to hypertensive patients. Our study found that only a small percent of those with high blood pressure receive advice to reduce salt intake, which can significantly improve blood pressure control, particularly in those with hypertension. It must be acknowledged however, that it is difficult for individuals to reduce dietary salt intake when more than 70% of the salt consumed is already present in the food supply and a general reduction in the salt content of manufactured foods is needed to effect a population wide reduction in blood pressure[30].

It is likely that GPs have little time during a single consultation to dedicate to lifestyle advice in one consultation. In addition, there are limited options available to overweight patients for assistance with weight loss. A multi-disciplinary resource that GPs could refer relevant patients may be one way ahead. Research should also focus on developing effective lifestyle interventions that GPs could deliver briefly, for those times where GPs are able to dedicate a few minutes to lifestyle advice. Strategies should also be investigated to encourage GPs to assess risk of overweight/obesity and that would support GPs in providing simple advice to assist patients in making positive lifestyle changes, which may assist in reducing weight gain in the general population. One such strategy is a national initiative called lifescripts that aims to assist GPs in offering lifestyle advice for risk factor management including weight management[31]. However, there still needs to be some form of motivation to encourage GPs to effectively utilise such initiatives.

## List of abbreviations

GP: General Practitioner.

## Competing interests

The authors have no conflict of interest to declare. Funding was obtained from the School of Exercise and Nutrition Sciences and the Centre for Physical Activity and Nutrition, Deakin University.

## Authors' contributions

AOB and CAN both contributed to the development of the questions for the survey interpreting the results and revising the manuscript. AOB was responsible for the statistical analyses of the data and writing of the initial draft of the manuscript. AOB and CAN read and approved the final manuscript.

## Pre-publication history

The pre-publication history for this paper can be accessed here:

http://www.biomedcentral.com/1471-2296/11/8/prepub
